# Clinical predictors of propranolol responsiveness in pediatric migraine: a prospective observational study

**DOI:** 10.22514/jofph.2026.026

**Published:** 2026-03-12

**Authors:** Müge Baykan, Elif Didinmez Taşkırdı, Özge Baykan Çopuroğlu, Pınar Gençpınar, Nihal Olgaç Dündar

**Affiliations:** ^1^Department of Pediatric Neurology, Recep Tayyip Erdoğan University, Rize Training and Research Hospital, 53100 Rize, Turkey; ^2^Department of Pediatric Neurology, Izmir City Hospital, 35530 Izmir, Turkey; ^3^Department of Therapy and Rehabilitation, Kayseri University, 38280 Kayseri, Turkey; ^4^Department of Pediatric Neurology, Izmir Katip Çelebi University, 35620 Izmir, Turkey; ^5^Neuroscience Center, Izmir Katip Çelebi University, 35620 Izmir, Turkey

**Keywords:** Pediatric migraine, Propranolol, Behavioral therapy, PedMIDAS, Personalized treatment

## Abstract

**Background**: This study aimed to evaluate the comparative effectiveness of propranolol therapy and structured behavioral interventions in reducing headache severity in pediatric patients and to identify predictors of treatment response. **Methods**: In this prospective, single-center study, 178 pediatric patients diagnosed with migraine based on the International Classification of Headache Disorders, 3rd edition (ICHD-3) criteria were enrolled. Participants were allocated into two groups according to baseline Pediatric Migraine Disability Assessment Scale (PedMIDAS) scores: Group 1 (PedMIDAS <15, n = 88) received standardized behavioral therapy, while Group 2 (PedMIDAS ≥15, n = 90) received propranolol (1–3 mg/kg/day) for 12 weeks. Primary outcomes were predefined as changes in monthly migraine attack frequency, PedMIDAS scores, and Visual Analog Scale (VAS)-measured headache intensity. Vitamin D deficiency and vitamin B_12_ deficiency were evaluated as biochemical predictors, and adherence was monitored bi-weekly. **Results**: Both groups showed significant improvement at week 12. Monthly migraine attacks declined from 3.5 ± 1.6 to 2.1 ± 1.2 in Group 1 and from 6.4 ± 2.1 to 3.1 ± 1.7 in Group 2. PedMIDAS scores decreased from 8.60 ± 3.25 to 5.75 ± 2.52 and 24.40 ± 9.65 to 16.11 ± 7.72, respectively (*p* < 0.001 both). VAS scores also improved in both groups with no significant between-group difference in percentage reduction. A ≥50% reduction in attack frequency plus ≥1-grade PedMIDAS improvement defined treatment response. In the propranolol group, response was independently associated with benign paroxysmal vertigo and essential tremor, while vitamin D and vitamin B_12_ deficiency predicted poorer outcomes. **Conclusions**: Both propranolol and structured behavioral therapy effectively reduce migraine-related disability and pain in pediatric patients, yielding comparable proportional improvements. The identification of key clinical and biochemical predictors supports a personalized treatment approach, integrating comorbidity screening and nutritional assessment to optimize outcomes. **Clinical Trial Registration**: ClinicalTrials.gov/NCT07180043, retrospectively registered.

## 1. Introduction

Headache is one of the most frequently reported somatic complaints among children and adolescents, affecting up to 60% of the pediatric population at some point during development [1]. Although often benign, migraine—including migraine and tension-type headache—can significantly impair a child’s daily functioning, academic performance, and overall quality of life [2]. Unlike secondary headaches, which are attributed to identifiable underlying causes such as infections, trauma, or structural anomalies, primary headaches arise without an evident organic etiology and are typically recurrent in nature [3].

Among primary headaches, migraine is particularly a concern due to its debilitating symptoms, such as throbbing pain, photophobia, phonophobia, nausea, and in some cases, aura [4]. Pediatric migraine differs from its adult counterpart in both symptomatology and diagnostic complexity. Younger children may present with bilateral pain, shorter attacks, and more frequent gastrointestinal symptoms, making accurate diagnosis challenging [5]. To address these challenges, the International Classification of Headache Disorders, 3rd edition (ICHD-3), provides pediatric-specific diagnostic criteria to enhance consistency in clinical and research settings [6].

Gender-specific patterns also emerge across the pediatric lifespan. Prior to puberty, boys are slightly more affected; however, after the onset of menarche, the prevalence in girls significantly surpasses that in boys, likely due to hormonal fluctuations and psychosocial stressors [7]. This gender disparity reinforces the importance of adopting personalized approaches to headache management in children.

The treatment of pediatric migraine is multifaceted, encompassing pharmacologic, behavioral, and lifestyle interventions. First-line treatment typically includes non-pharmacologic strategies such as sleep hygiene, stress management, dietary modifications, and behavioral therapies. Recent pediatric guidelines emphasize that pharmacological prophylaxis should be reserved for children with moderate-to-severe migraine-related disability, while early initiation of drug therapy in patients with minimal impairment (*e.g.*, PedMIDAS <15) may result in unnecessary medication exposure and reduced adherence [8]. Therefore, non-pharmacological therapy remains the cornerstone of treatment in mildly affected patients, while pharmacologic agents are considered when functional impairment persists despite lifestyle modification.

Among the limited pharmacologic options, propranolol—a non-selective β-adrenergic blocker—remains one of the most commonly prescribed medications for pediatric migraine prevention [9, 10]. It exerts therapeutic effects through modulation of cortical spreading depression, attenuation of thalamocortical excitability, and regulation of autonomic tone, mechanisms that may be particularly relevant in children with comorbid autonomic dysregulation such as palpitations, anxiety, or essential tremor [11].

Although propranolol has long been established as effective for migraine prophylaxis in adults, evidence in pediatric populations remains limited and heterogeneous [12]. Previous studies have primarily evaluated the drug under controlled experimental settings. However, these settings do not fully reflect clinical decision-making, where treatment selection is influenced by baseline disability, family preferences, and ethical concerns regarding placebo use in children [13, 14].

Behavioral therapies, including cognitive behavioral training, relaxation techniques, and biofeedback, are widely recognized as beneficial adjuncts or alternatives to pharmacologic approaches, particularly for children with mild disability. Yet, few studies have directly compared structured behavioral therapy with pharmacologic treatment while stratifying patients based on baseline PedMIDAS severity. Clarifying this distinction is essential, as inappropriate use of pharmacotherapy in low-disability patients may contradict best-practice recommendations.

In addition to therapeutic modality, potential predictors of response such as comorbid psychiatric and neurological disorders (*e.g.*, anxiety, attention-deficit/hyperactivity disorder, benign paroxysmal vertigo (BPV), essential tremor) and micronutrient deficiencies (vitamin D and B_12_) may influence treatment outcomes [15, 16, 17]. Deficiencies in these vitamins have been linked to altered neuronal excitability, inflammation, and pain sensitization [18, 19]. Therefore, the integration of clinical, psychological, and biochemical markers may help optimize treatment selection, improve adherence, and prevent unnecessary pharmacologic exposure.

Accordingly, the present study aimed to assess the effectiveness of propranolol compared with structured behavioral therapy in reducing migraine-related disability and pain among pediatric patients, while identifying clinical, psychiatric, and biochemical predictors of treatment responsiveness.

## 2. Materials and methods

### 2.1 Study design and setting

This study was designed as a prospective comparative observational study conducted between January 2021 and December 2023 at the Pediatric Neurology Department of a tertiary referral center. Ethical approval was obtained from the Tepecik Training and Research Hospital Ethics Committee (Approval No: 2021/12-14), and the study adhered to the principles of the Declaration of Helsinki. The trial was retrospectively registered at ClinicalTrials.gov (NCT07180043). Written informed consent was obtained from the legal guardians of all participants prior to enrollment.

### 2.2 Participants and eligibility criteria

A total of 178 children, aged between 6 and 16 years, diagnosed with migraine based on the ICHD-3 criteria, were included. Participants were consecutively recruited from the pediatric neurology outpatient clinic. The inclusion criteria required a confirmed diagnosis of migraine with or without aura according to ICHD-3 criteria, a minimum migraine frequency of four attacks per month, and no history of prophylactic migraine therapy within the last three months.

Patients were excluded if they had secondary headaches due to underlying pathologies such as tumors, infections, or vascular malformations, chronic systemic or psychiatric disorders including epilepsy and major depression, contraindications to propranolol such as asthma or cardiac conduction defects, or incomplete clinical data or follow-up. Psychiatric disorders in the exclusion criteria referred specifically to major psychiatric conditions (*e.g.*, major depressive disorder, bipolar disorder, psychosis, severe obsessive-compulsive disorder) or any psychiatric illness requiring pharmacological treatment or hospitalization. In contrast, mild-to-moderate anxiety symptoms or social phobia without functional impairment or medication requirement were not considered exclusionary. These conditions were included as clinical comorbidities frequently observed in pediatric migraine and were systematically evaluated as potential predictors of treatment response.

A power analysis was performed using G*Power 3.1 (Heinrich Heine University Düsseldorf, Düsseldorf, NRW, Germany) to estimate the required sample size. Assuming an effect size of 0.35, a significance level of α = 0.05, and a statistical power of 0.90, the minimum required sample size was calculated to be 160 participants. To account for potential dropout, a total of 178 children were recruited (Fig. 1).

**Fig. 1.** CONSORT flow diagram of participant enrollment and follow-up. PedMIDAS: Pediatric Migraine Disability Assessment Scale.

### 2.3 Baseline assessment and treatment allocation

At baseline, all participants underwent detailed clinical evaluation, including demographic characteristics, migraine history, monthly migraine attack frequency, Pediatric Migraine Disability Assessment (PedMIDAS) scores, and average headache intensity over the previous month using the Visual Analog Scale (VAS, 0–10). Laboratory analyses included serum vitamin D, vitamin B_12_, and homocysteine levels.

Participants were not randomized; rather, allocation was based on baseline PedMIDAS severity to ensure ethical treatment selection. Children with mild disability (PedMIDAS <15) were assigned to structured behavioral therapy (Group 1), while those with moderate-to-severe disability (PedMIDAS ≥15) received propranolol (Group 2). This stratification is in accordance with pediatric migraine guidelines recommending conservative, non-pharmacological management for patients with minimal disability and reserving pharmacologic prophylaxis for those with significant functional impairment.

Importantly, all participants—regardless of group—received standard non-pharmacological counseling at baseline, including advice on regular sleep routines, hydration, trigger avoidance (such as skipped meals, excessive screen time, or caffeine), stress management, and appropriate acute analgesic use.

Group 1 additionally participated in a structured behavioral intervention program consisting of scheduled headache education sessions, sleep hygiene coaching, relaxation and breathing techniques, and maintenance of a headache diary supervised by a clinician. No preventive medication was prescribed in this group.

Group 2 received oral propranolol, initiated at 1 mg/kg/day and titrated every two weeks up to a maximum of 3 mg/kg/day, depending on clinical response and tolerability (median effective dose 2.2 mg/kg/day; range 1.5–3.0 mg/kg/day). Aside from routine lifestyle advice provided at baseline, no structured behavioral sessions were administered in this group to avoid treatment overlap and to maintain clear comparison between modalities.

### 2.4 Follow-up and outcome measures

Participants were evaluated at baseline and subsequently at weeks 4, 8, and 12 after the initiation of treatment. At each visit, clinical data were recorded, including monthly migraine attack frequency, headache duration, associated symptoms, and treatment adherence. Migraine-related disability was assessed using the Pediatric Migraine Disability Assessment Scale (PedMIDAS), and headache intensity was evaluated using the Visual Analog Scale (VAS; 0–10), reflecting the average pain intensity over the preceding month.

In this study, migraine attack frequency, PedMIDAS scores, and VAS headache intensity were designated as primary outcome measures. Changes from baseline to week 12 were examined in both absolute values and percentage reduction. In addition, patients were classified based on treatment response. A responder was defined as a participant who demonstrated at least a 50% reduction in monthly migraine frequency together with an improvement of at least one PedMIDAS grade compared with baseline. Participants who did not meet these criteria were classified as non-responders.

Venous blood samples were collected at baseline and again at the 12-week visit. Baseline serum levels of vitamin D, vitamin B_12_, and homocysteine were used for predictive modeling, as follow-up measurements could have been influenced by dietary supplementation or seasonal variation during the intervention period. Vitamin D deficiency was defined as <20 ng/mL and insufficiency as 20–30 ng/mL; vitamin B_12_ deficiency was defined as <200 pg/mL and insufficiency as 200–300 pg/mL; and elevated homocysteine was defined as >15 μmol/L. Laboratory analyses were performed using validated enzyme-linked immunosorbent assay (ELISA) kits by technicians blinded to treatment allocation.

### 2.5 Statistical analysis

All statistical analyses were performed using IBM SPSS Statistics version 28.0 (IBM Corp., Armonk, NY, USA). Data were first examined for normality using the Shapiro-Wilk test and for homogeneity of variances using Levene’s test. Continuous variables were expressed as mean ± standard deviation (SD) for normally distributed data and median (interquartile range, IQR) for non-normally distributed data, while categorical variables were presented as frequencies and percentages.

Changes in migraine attack frequency, PedMIDAS scores, and VAS headache intensity from baseline to week 12 were evaluated as both absolute and percentage differences. Percentage change was calculated for each participant using the formula: [(baseline value − week 12 value)/baseline value] × 100. Within-group comparisons were conducted using paired *t*-tests for normally distributed variables or the Wilcoxon signed-rank test for non-normally distributed data. Between-group differences were analyzed using independent samples *t*-tests or the Mann-Whitney U test, as appropriate. Categorical data were compared using the Chi-square test or Fisher’s exact test.

Given that three co-primary outcomes were analyzed (migraine frequency, PedMIDAS, and VAS scores), the significance threshold was adjusted for multiple testing using a Bonferroni correction. Effect sizes were calculated using Cohen’s *d* for continuous variables and reported to aid interpretation of clinical relevance.

To identify independent predictors of response to propranolol treatment, univariate analyses were initially performed. Variables with a *p*-value < 0.10 were subsequently entered into a multivariable binary logistic regression model, with treatment response (responder vs non-responder) as the dependent variable. Independent variables included benign paroxysmal vertigo, essential tremor, anxiety, social phobia, vitamin D deficiency, and vitamin B_12_ deficiency. Model fit was assessed using the Hosmer-Lemeshow goodness-of-fit test, and results were reported as adjusted odds ratios (ORs) with 95% confidence intervals (CIs). Predictive performance of significant variables was evaluated using Receiver Operating Characteristic (ROC) curve analysis, and the area under the curve (AUC) was used to determine discriminative accuracy. Multicollinearity was checked using the Variance Inflation Factor (VIF), and values <3 were considered acceptable.

Missing data were minimal (<3%) and were handled using multiple imputations with five iterations under the assumption of missing at random (MAR). All analyses were conducted on a per-protocol basis. All tests were two-tailed, and a *p*-value < 0.05 was considered statistically significant.

## 3. Results

A total of 178 pediatric migraine patients were enrolled in the study. The mean age was 11.98 ± 4.8 years (range: 6–16), and 59.6% (n = 106) were female. Among them, 93 patients (52.2%) were diagnosed with migraine without aura, 10 patients (5.6%) had migraine with aura, and 75 patients (42.1%) had mixed-type headache. Of those with aura, six patients reported visual aura and four reported paresthesia. There were no significant differences between groups regarding age, sex, or migraine subtype (*p * > 0.05) (Table 1).

**Table 1.** Demographic and clinical characteristics of patients.

Characteristics	All Patients (n = 178)	Group 1 (n = 88)	Group 2 (n = 90)	*p*
Age (yr), Mean ± SD	11.98 ± 4.8	11.65 ± 4.9	12.31 ± 4.7	0.317
Female (%)	106 (59.6%)	53 (60.2%)	53 (58.9%)	0.861
Migraine w/o aura	93 (52.2%)	47 (53.4%)	46 (51.1%)	0.774
Migraine w/ aura	10 (5.6%)	4 (4.5%)	6 (6.7%)	0.537
Mixed-type headache, n (%)	75 (42.1%)	37 (42.0%)	38 (42.2%)	0.982

*w/o without; w/: with; SD: standard deviation.*

Monthly migraine attack frequency significantly decreased in both groups by week 12. In Group 1, the mean frequency declined from 3.5 ± 1.6 to 2.1 ± 1.2 attacks/month; in Group 2, from 6.4 ± 2.1 to 3.1 ± 1.7 attacks/month. Percentage reduction in attack frequency was 40.0% in Group 1 and 52.0% in Group 2; however, this difference was not statistically significant after baseline adjustment (*p* = 0.118). PedMIDAS scores significantly improved in both groups. Group 1 showed a reduction from 8.60 ± 3.25 to 5.75 ± 2.52, and Group 2 from 24.40 ± 9.65 to 16.11 ± 7.72. Percentage improvement in PedMIDAS was comparable (33.1% *vs.* 33.9%, *p* = 0.526). VAS scores also decreased in both groups (3.35 → 2.20 in Group 1; 4.40 → 3.10 in Group 2), with no significant between-group difference in percentage reduction (34.3% *vs. *29.5%, *p* = 0.289) (Table 2).

**Table 2.** Primary outcomes at baseline and week 12.

Outcome Measure	Group 1—Behavioral (n = 88)	Group 2—Propranolol (n = 90)	% Change [(baseline − week 12)/baseline] × 100	*p*
Monthly migraine attacks	3.5 ± 1.6 → 2.1 ± 1.2	6.4 ± 2.1 → 3.1 ± 1.7	40.0% *vs.* 52.0%	0.118^†^
PedMIDAS score	8.60 ± 3.25 → 5.75 ± 2.52	24.40 ± 9.65 → 16.11 ± 7.72	33.1% *vs.* 33.9%	0.526
VAS headache intensity	3.35 → 2.20	4.40 → 3.10	34.3% *vs.* 29.5%	0.289

*^†^Attack frequency comparison adjusted for baseline using percentage change method (% change = [(baseline − week 12)/baseline] × 100). PedMIDAS: Pediatric Migraine Disability Assessment Scale; VAS: Visual Analog Scale.*

Treatment response was evaluated using predefined criteria, including a ≥50% reduction in monthly migraine attack frequency and at least one-grade improvement in PedMIDAS disability classification at week 12. In the behavioral therapy group, 33 out of 88 patients (37.5%) met the responder criteria. In the propranolol group, 55 out of 90 patients (61.1%) were classified as responders. Patients who did not meet either threshold were categorized as non-responders. No treatment discontinuations or protocol deviations occurred during the study period.

Within the propranolol group, univariate analysis showed that benign paroxysmal vertigo (BPV), essential tremor, social phobia, and anxiety were more frequent among responders, while vitamin D and vitamin B_12_ deficiencies were more common among non-responders. Multivariable logistic regression identified BPV (OR = 3.82, 95% CI: 2.11–6.93) and essential tremor (OR = 3.27, 95% CI: 1.89–5.66) as independent positive predictors of treatment response. Vitamin D deficiency (OR = 0.54, 95% CI: 0.33–0.89) and vitamin B_12_ deficiency (OR = 0.48, 95% CI: 0.28–0.82) were independent negative predictors. Parental migraine history was not significantly associated with treatment success (Table 3).

**Table 3.** Factors associated with propranolol treatment response.

Predictor	Responders (n = 55)	Non-responders (n = 35)	Univariate OR (95% CI)	*p*	Multivariable OR (95% CI)	*p*
BPV	45 (81.8%)	5 (14.3%)	4.12 (2.20–7.71)	<0.001	3.82 (2.11–6.93)	<0.001
Essential tremor	37 (67.3%)	8 (22.9%)	3.54 (1.95–6.42)	<0.001	3.27 (1.89–5.66)	<0.001
Social phobia	25 (45.5%)	9 (25.7%)	2.05 (1.13–3.72)	0.018	1.88 (1.12–3.21)	0.017
Anxiety	35 (63.6%)	13 (37.1%)	1.52 (1.01–2.34)	0.046	1.41 (0.93–2.14)	0.089
Vitamin D deficiency (<20 ng/mL)	15 (27.3%)	41 (77.8%)	0.57 (0.35–0.92)	0.021	0.54 (0.33–0.89)	0.004
Vitamin B_12_ deficiency (<200 pg/mL)	17 (30.9%)	39 (74.3%)	0.49 (0.29–0.82)	0.006	0.48 (0.28–0.82)	0.002
Parental migraine	48 (87.3%)	42 (85.7%)	1.08 (0.67–1.73)	0.735	-	-

*BPV: Benign Paroxysmal Vertigo; OR: odds ratios; CI: confidence interval.*

ROC analysis demonstrated that BPV had the highest predictive accuracy for propranolol response, with an AUC of 0.871 (95% CI: 0.812–0.916). Essential tremor also showed strong discriminative capacity (AUC = 0.812). Vitamin B_12_ deficiency (AUC = 0.789) and vitamin D deficiency (AUC = 0.742) demonstrated moderate predictive value. Sensitivity and specificity rates for all predictors are provided in Table 4.

**Table 4.** ROC curve analysis of predictors of propranolol treatment response.

Predictor	AUC	95% CI	Sensitivity (%)	Specificity (%)
BPV	0.871	0.812–0.916	83.6	79.2
Essential Tremor	0.812	0.744–0.868	78.1	75.4
Vitamin B_12_ Deficiency	0.789	0.715–0.849	74.6	72.8
Vitamin D Deficiency	0.742	0.664–0.809	69.2	70.3

*BPV: Benign Paroxysmal Vertigo; ROC: Receiver Operating Characteristic; AUC: Area Under the Curve; CI: Confidence Interval.*

Adverse events were observed only in the propranolol group. A total of nine patients (10%) reported mild and transient side effects, including fatigue (n = 5, 5.6%), dizziness (n = 3, 3.3%), and asymptomatic bradycardia (n = 1, 1.1%). No serious adverse events, treatment discontinuations, or hospitalizations occurred during the 12-week study period. No adverse effects were reported in the behavioral therapy group, and all participants completed the study as per protocol.

## 4. Discussion

This prospective, comparative observational study evaluated the real-world clinical effectiveness of propranolol and structured behavioral therapy in pediatric migraine and identified clinical and biochemical predictors of treatment response. Treatment allocation was based on baseline PedMIDAS severity rather than randomization to avoid unnecessary pharmacotherapy in children with minimal disability, in accordance with current pediatric headache guidelines advocating non-pharmacological approaches—such as sleep hygiene, stress regulation, and lifestyle modification—as first-line interventions in mild migraine [3, 8, 12]. This pragmatic design reflects real-life clinical decision-making and enhances external validity, although it inherently limits causal inference.

Both behavioral therapy and propranolol led to significant reductions in monthly migraine frequency, headache intensity, and PedMIDAS scores, demonstrating that stratified, disability-based treatment selection can achieve clinically meaningful outcomes across severity levels. Although patients receiving propranolol had more severe baseline disability by design, percentage reductions in PedMIDAS and VAS were comparable between groups, supporting individualized treatment rather than uniform prophylactic administration.

Propranolol demonstrated robust clinical efficacy and safety, consistent with previous pediatric trials [7, 10, 11, 12, 20]. Its therapeutic effect is thought to arise from modulation of β-adrenergic tone, stabilization of cortical excitability, and restoration of autonomic balance [9, 21, 22]. Conversely, structured behavioral therapy—which included education, stress regulation, sleep management, and diary-based self-monitoring—was effective in patients with mild disability, reinforcing guideline recommendations that behavioral interventions should be prioritized before pharmacotherapy in low-burden migraine.

Several baseline clinical features were independently associated with better response to propranolol, particularly benign paroxysmal vertigo (BPV), essential tremor, social phobia, and anxiety [23, 24, 25]. These comorbidities suggest a shared pathophysiological mechanism involving cerebello-thalamocortical dysregulation, autonomic hyperexcitability, and increased sympathetic output [26, 27]. Propranolol’s β-blocking properties may attenuate these mechanisms through modulation of Purkinje cell activity and central noradrenergic networks, thereby improving response in this subgroup [27, 28].

Vitamin D and B_12_ deficiencies emerged as significant negative predictors of treatment response. These findings are biologically plausible, given that vitamin D modulates neuroinflammation and calcium homeostasis, while vitamin B_12_ deficiency increases homocysteine, contributing to endothelial dysfunction and impaired neuronal signaling [16, 17, 18, 19, 29, 30, 31]. Thus, baseline micronutrient assessment may help identify children who require concurrent supplementation to optimize prophylactic efficacy.

The predictive model demonstrated strong discriminative accuracy (AUC >0.80 for BPV and essential tremor) and acceptable calibration, supporting the utility of integrating neurological comorbidities and biochemical markers in treatment planning. This precision-based approach aligns with contemporary shifts in pediatric migraine management toward personalized care [29, 32, 33].

Propranolol was well tolerated, with only mild fatigue, dizziness, and transient bradycardia reported—none requiring treatment withdrawal—consistent with previous safety data [10, 12, 22]. High adherence rates in both groups (>80%) strengthen the reliability of observed outcomes.

This study has several strengths, including its prospective design, predefined outcomes, high adherence, and combined evaluation of clinical, psychological, and biochemical predictors. However, limitations include the non-randomized design, which may introduce selection bias and confounding, and the 12-week follow-up period, which does not allow assessment of long-term recurrence or sustained remission. Additionally, behavioral therapy effectiveness may vary depending on patient engagement and familial support.

Overall, the findings reinforce that treatment selection in pediatric migraine should be individualized based on clinical severity, comorbidities, and micronutrient status. Behavioral therapy is appropriate for mild cases, whereas propranolol is effective in patients with greater disability or neurological comorbidities when micronutrient levels are adequate. Incorporating clinical, psychological, and biochemical markers at baseline may optimize treatment efficacy, enhance patient satisfaction, and avoid unnecessary medication exposure.

In conclusion, both propranolol and structured behavioral therapy effectively reduced migraine burden in children. Propranolol responsiveness was independently predicted by BPV, essential tremor, social phobia, and anxiety, whereas vitamin D and B_12_ deficiencies were associated with diminished response. These results support an integrated, personalized treatment model that combines pharmacological, behavioral, and nutritional strategies to improve clinical outcomes and quality of life in pediatric migraine.

## 5. Conclusions

This study demonstrates that both propranolol and structured behavioral therapy effectively reduce migraine-related disability and pain severity in pediatric patients. Although propranolol showed slightly greater absolute improvements, proportional reductions in PedMIDAS and VAS scores were comparable, supporting the clinical value of behavioral therapy as a first-line approach.

The identification of predictors—such as benign paroxysmal vertigo, essential tremor, anxiety traits, and vitamin D/B_12_ status—highlights the potential for individualized treatment planning. Future multicenter, randomized studies are warranted to validate these findings and to guide precision-based strategies for optimizing pediatric migraine management.
